# Improving patient outcomes: leveraging data to drive innovation in health care – New South Wales’ Activity-Based Funding management portal

**DOI:** 10.1186/1472-6963-15-S2-A1

**Published:** 2015-06-15

**Authors:** Alfa Damato

**Affiliations:** 1 New South Wales Health / Activity-Based Funding Taskforce, Ministry of Health, North Sydney, New South Wales, 2060, Australia

## Background

Clinical and non-clinical health leaders both seek to leverage data to drive innovative improvements in models of care and create sustainable and effective health systems. For the last three years, Activity-Based Funding (ABF) has been the main driver for funding public hospitals in New South Wales (NSW), Australia. The data generated by the implementation of ABF has been used for both policy and funding decisions. As quality improved, the data became more relevant to a wide range of stakeholders, and drove improved interactions and conversations between clinicians and administrators.

These conversations and opportunities were particularly relevant for patient-level clinical costing. This process uses a large range of data sets to allocate costs, and is presented with patient activity data that are familiar to clinicians. Clinical analytics, which leverages on cost and activity data, are being used to shape the future of local health systems, improve service delivery, and enhance patient outcomes. This shift moves the system from ABF to Activity-Based Management (ABM), through which data can be used not only for funding the annual budgets of Local Health Districts (LHDs) but also to inform local policy decisions.

For several years, LHDs have submitted annual patient costing data returns to the Ministry of Health. Until recently, however, they saw few benefits from the effort required to submit these data, since state-wide benchmarking and variance analysis were limited and/or provided with significant time delays. The ABM Portal was created and launched to address this issue.

The Portal provides a rich data source that can support local decision-making about clinical care evaluations, reduce unwarranted clinical variations, improve care models, facilitate service planning, and effectively manage services within budget. The ABM Portal was developed with significant input from clinicians and is currently being expanded to incorporate additional data elements. The ABM Portal contains data aggregated to LHDs from patient-level data, enabling users to drill down to the lowest-level information and understand the causes of any identified variances (i.e., in cost or length of stay).

## Materials and methods

A number of steps were employed to support the health system’s transition to ABM, and use of the ABM Portal. These included redesigning processes to provide data to the Ministry of Health, establishing data quality assurance processes, and establishing clinical engagement strategies.

Redesigning processes focused on streamlining and merging a number of data collections to enable a single submission to be used for multiple purposes. Following the process redesign, significant effort was invested in improving data quality and accessibility to stakeholders, thereby creating a supportive environment for data improvement exercises.

The final, critical step was designing data associations and relationships that are meaningful for clinicians and maintain a bridge with the administrators. In addition to benchmarking at the hospital level, the change to ABM enabled the system to focus on patients.

## Results

The move to ABM has fostered a significant cultural change in NSW Health, driven by the transparency of information contained in the ABM Portal. Stakeholders can now access information about their own and their peers’ services in a more timely manner. The level of detail contained in the ABM Portal enables users to test common hypothesis about their services, such as whether their patients are older, sicker, and/or more complex, compared to their peers’ patients. As a result, performance issues can be unbundled further, in order to identify core issues that drive costs and establish a clear relationship between all domains of performance including those that are activity-related and financial.

Users can access comprehensive information about the “Patient Journey” via the ABM Portal. Because the information contained in the ABM Portal is at the patient level, users can easily identify the most frequent patients, analyze their journey through the system, and assess the treatments received and their associated costs in a variety of settings. This has facilitated a shift from traditional case-mix approaches, which are anchored in care type, to a more holistic and patient-centered health care system.

This shift has been critical to implementing changes in models of care that improve patient outcomes. It has supported a more effective and productive planning from the clinical and non clinical point of views, as well as the establishment of a more expansive and integrated health care system.

Users are now empowered and can find answers to their questions, rather than having to request a report and wait several weeks for it to be prepared. This process has improved the efficiency of sourcing and using relevant organizational information, and is driving changes across the health care system.

## Conclusions

As Australian health care budgets tighten, and more is learned about the impact of ABF, clinicians and managers increasingly rely on data. Yet, this reliance requires suitable tools and appropriately skilled and experienced staff to manage large, complex health information repositories, and thereby drive changes across the health care system.

The ABM Portal is an important step to help create a sustainable health care system that uses innovative care models to improve patient outcomes. The Portal’s development has been successful in an economic climate of high urgency and low funding availability.

The ABM Portal solution jump-started NSW Health’s use of data. The Portal places patient care at a system grounded in data that are easily used to support clinical and administrative decisions, transparent, and focused on a comprehensive health care service model. The AMB Portal’s “Patient Journey” feature is a clear example of how complex data can support local clinical and non-clinical decision-making. This development is leading to more integrated care systems that will have a positive impact on patient outcomes.

